# 

**Published:** 1916-08

**Authors:** 


### Contents

Event and Comment . . . . . . . . . . . . 351

The Therapeutic Efficiency of Oral Preparations . . . . . . . . . . . . 353

The Interproximal Space and the Contact Point . . . . . . . . . . . . 366

Treatment of Pyorrhœa by the General Practitioner . . . . . . . . . . . . 377

The Indirect or Model Method for Inlays and Crowns . . . . . . . . . . . . 386

Nitrous Oxide the Safest Volatile Anesthetic . . . . . . . . . . . . 390

Indents . . . . . . . . . . . . 391

Announcements . . . . . . . . . . . . 399

